# GEFA-YOLO: Lightweight Weed Detection with Group-Enhanced Fusion Attention

**DOI:** 10.3390/s26020540

**Published:** 2026-01-13

**Authors:** Huicheng Li, Pushi Zhao, Feng Kang, Yuting Su, Qi Zhou, Zhou Wang, Lijin Wang

**Affiliations:** 1 College of Computer and Information Science, Fujian Agriculture and Forestry University, Fuzhou 350002, China; 52411049022@fafu.edu.cn (H.L.); 52511049046@fafu.edu.cn (P.Z.); 52411049019@fafu.edu.cn (F.K.); 52411049039@fafu.edu.cn (Y.S.); zq200306@163.com (Q.Z.); 52311049016@fafu.edu.cn (Z.W.); 2 Key Laboratory of Smart Agriculture and Forestry, Fujian Province University, Fuzhou 350002, China

**Keywords:** object detection, attention mechanism, group-enhanced fusion attention, convolutional neural networks, weed detection system

## Abstract

Cotton is an important economic crop, and its weed management directly affects yield and quality. In actual cotton fields, detection accuracy still faces challenges due to the complex types of weeds, variable morphologies, and environmental factors. Most existing models rely on the attention mechanism to improve performance, but channel attention tends to ignore spatial information, while full spatial attention brings high computational costs. Therefore, this paper proposes a grouped enhanced fusion attention mechanism (GEFA), which combines grouped convolution and local spatial attention to reduce complexity and parameter quantity while effectively enhancing feature expression ability. The GEFAY detection model constructed based on GEFA achieves good balance in efficiency, accuracy, and complexity on the CottonWeedDet12, VOC, and COCO datasets. Compared with classic attention methods, this model has the smallest increase in parameters and computational costs while significantly improving accuracy. It is more suitable for deployment on edge devices. The further designed end-to-end intelligent weed detection system and edge device deployment can achieve image detection on local maps and real-time cameras, with good practicality and scalability, providing effective technical support for intelligent visual applications in precision agriculture.

## 1. Introduction

With the transformation of modern agriculture toward precision and automation, the importance of weed identification technology in agricultural field management has become increasingly prominent, especially in the complex identification of weeds in fields, where a series of unique challenges are faced. Cotton, as an economic crop, is highly susceptible to interference from weeds during its growth process, which not only affects the growth and development of cotton but also may increase the amount of pesticide use, causing environmental pollution and resource waste [1]. In recent years, convolutional neural networks (CNN) have made significant progress in thefield of image recognition and have been widely applied in tasks such as target detection. For example, R-CNN [2] achieves precise detection through region proposal and deep feature learning, while models like YOLO [3] and SSD [4] can achieve fast end-to-end object detection. With the rapid development of object detection technology, it is now applied in various important fields, including pedestrian and vehicle detection in autonomous driving [5,6], retail product recognition [7,8], public safety monitoring [9], and smart agriculture [10,11]. Although CNN effectively reduces the number of network parameters through local receptive fields and weight sharing, it still has the problem of receptive field limitation, which cannot well capture global features. This is particularly important for weed detection in cotton fields, as weed types are complex and their shapes are variable, often sharing similar colors and textures with the crops, with complex backgrounds and uneven distribution of weeds, resulting in insufficient performance of the network when processing such complex backgrounds. Moreover, in actual agricultural production scenarios, the weed recognition module is often deployed on agricultural machinery platforms, field inspection equipment, or embedded terminals. Its computing resources, storage space, etc., are all subject to strict limitations. To address this issue, it is necessary to enhance the model’s expression ability and improve the performance of the detection model within the limited computing budget by adopting more efficient feature modeling methods.

To enhance the feature representation capability, the attention mechanism has been introduced to improve the performance of the model. This mechanism simulates the characteristics of human visual systems, focusing on key areas, enabling the network to allocate resources more effectively when processing images. With this mechanism, the network can more accurately capture key information in the image and, to a certain extent, possess the advantage of capturing long-range dependencies. For example, He et al. [12] proposed the EDS-YOLOv8 model, which integrates EfficientViT, dynamic snake convolution, and SimAM attention mechanism, significantly improving the detection accuracy of dense and needle-like weeds by 6.4% at mAP@0.5; Zhang et al. [13] proposed the SE-YOLOv5x model, which embedded an SE attention module at the end of the YOLOv5x backbone, adaptively re-calibrated channel features, and significantly enhanced the expression ability of subtle textures of weed leaves; by connecting an SE attention module after the SPP layer and designing local importance pooling, Chen et al. [14] addressed the problem of missed detection due to the high similarity in color and texture between sesame plants and weeds in sesame fields to strengthen the subtle differences between sesame main stems and broad-leaved weeds, with an average precision mean (mAP) of 96.16%; Jing et al. [15] embedded Coordinate Attention into the cross-stage connection module of YOLOXs, balancing the relationships between channels and position information and achieving excellent average detection accuracy for oilseed rape and weeds in the field; Guo et al. [16] addressed the problem of chaotic background and large contrast in the lower parts of rice images for YOLOv8, integrating FPN + CBAM modules, improving by 4.8% compared to the original YOLOv8, maintaining a 55 FPS inference speed, and verifying the robustness of the attention mechanism in low-light, high-humidity paddy fields. However, most current methods often focus only on the modeling of channel or spatial features separately, failing to fully integrate the two; the channel attention mechanism can allocate channel weights by learning the distribution of feature information, thereby highlighting the information that deserves attention [17]. The attention mechanism optimizes the weight distribution between channels, improving the accuracy and overall performance of the model [18,19]. However, it ignores the importance of spatial information and cannot focus on spatial [20] details. Other channel attention mechanisms also have similar problems [21,22]. Introducing complex spatial modeling and high-dimensional feature interaction brings about a large number of parameters and computational costs. For instance, although CBAM [23] can effectively enhance the expression of channel and spatial features, its structure is complex and has high costs, which is not conducive to integration into lightweight models [24] and makes it difficult to meet real-time requirements on embedded platforms, thus limiting its application in actual agricultural scenarios. Optimizing CBAM usually involves reducing the number of channels to lower complexity, but this may lead to information loss. To address the problems existing in the current attention mechanisms, we propose a group-enhanced fusion attention mechanism (GEFA) with the following specific contributions.

1. Propose the GEFA attention mechanism, which enhances feature extraction capabilities through the combination of group convolution, one-dimensional convolution, and residual connections, effectively improving the comprehensive performance of the attention mechanism.
2. Propose a named group-enhanced fusion attention YOLO (GEFAY) object detection network, which integrates multiple attention mechanisms (such as SE, CBAM, mixed local channel attention (MLCA), etc.) and the proposed GEFA.
3. Experiments conducted on multiple datasets show that GEFA and GEFAY are lightweight and feasible.
4. The innovations in deployment and interaction ensure detection performance while supporting the deployment of embedded devices. It also designs a visualization output and interaction interface to solve the problem of “difficulty in implementation and operation” of traditional algorithms.

The structure of this paper is as follows: Section 2 introduces the principle and architecture of the GEFA algorithm. Section 3 describes the experimental design and results on multiple datasets. Section 4 is about the design and implementation of weed detection, and Section 5 is the conclusion.

## 2. Methodology

### 2.1. Attention Mechanism

The attention mechanism plays a crucial role in deep learning, significantly enhancing the model’s ability to handle complex tasks by focusing attention on the most critical parts of the input data. Based on its working principle, the attention mechanism can be roughly divided into a soft attention mechanism and a hard attention mechanism. The soft attention mechanism assigns a weight ranging from 0 to 1 to each input feature, enabling dynamic attention to the input information. This mechanism enables the model to comprehensively consider each feature during processing. However, due to the non-binary distribution of weights, the soft attention mechanism is relatively computationally expensive.

Typical examples of soft attention mechanisms include SE, CBAM, SK [25], and self-attention mechanism [26]. These methods enhance the model’s ability to capture the relationships between features in various ways. For example, the SE mechanism is an effective channel attention method that uses compression and excitation strategies to emphasize channel information. The SE module first compresses each channel using global average pooling and then adjusts the activation values of each channel through a fully connected layer, dynamically adjusting the channel weights. This mechanism significantly improves the model’s sensitivity to key channels, thereby enhancing overall performance. Additionally, the self-attention mechanism in the Transformer model is a classic application of the soft attention mechanism. Note. This mechanism calculates the dot product similarity between elements of the input sequence and applies the softmax function to obtain standardized weights, enabling the model to focus on the dynamic relationships between sequence elements. This allows the Transformer to capture long-range dependencies, significantly improving performance in tasks such as natural language processing. In visual Transformers, the cross-attention (CAT) mechanism alternately applies the attention mechanism between image regions and within the image to collect global information while enhancing local detail perception. This strategy of combining global and local information further enhances the model’s ability to handle the complexity of visual scenes. In contrast, the hard attention mechanism assigns binary weights (0 or 1) to input features, focusing only on the most important parts. This binary weight distribution significantly reduces the computational burden, making hard attention suitable for applications that are sensitive to computing time and cost. Representative hard attention techniques include Bayesian attention [27], Expectation Maximization attention [28], and Gaussian attention mechanism [29]. For example, the Bayesian attention mechanism selects key features precisely using a Bayesian model, the Expectation Maximization attention mechanism optimizes feature selection through the expectation maximization algorithm, and the Gaussian attention mechanism simplifies the feature selection process using a Gaussian model. In summary, the introduction of the attention mechanism greatly enriches the functionality of deep learning models, enabling them to process information more effectively in complex environments. Although the soft attention mechanism has a large computational cost, it can precisely capture and utilize the detailed information in the data. In contrast, the hard attention mechanism, due to its high computational efficiency, is well-suited for scenarios that require rapid responses. By appropriately selecting and applying these mechanisms, the performance and efficiency of the model can be optimized according to specific task requirements.

### 2.2. YOLOv5 Benchmark Model

**Figure 1:**
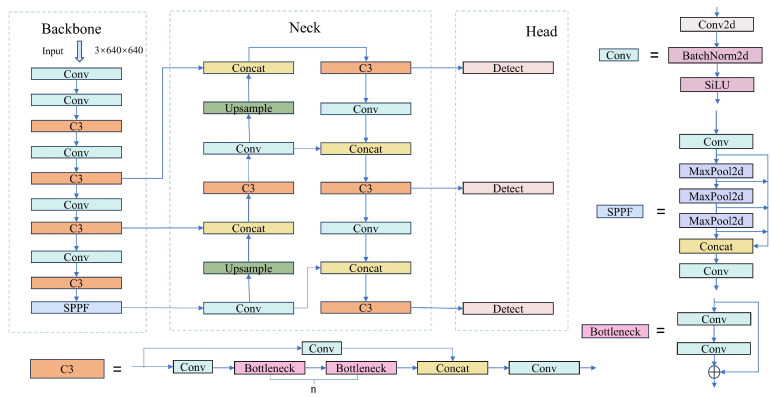
The YOLOv5 network architecture.

YOLOv5 (You Only Look Once v5) [30] is the fifth generation version of the YOLO series of object detection algorithms, widely used in real-time object detection tasks. Compared with previous models, YOLOv5 has optimized in terms of detection speed, accuracy, and engineering usability, and has become one of the mainstream models in the current object detection field. Its network structure mainly consists of four parts: the input end, the backbone network (Backbone), the neck network (Neck), and the detection head (Head). The input end introduces strategies such as Mosaic data augmentation, adaptive anchor box allocation, and adaptive image scaling to enhance the robustness and generalization ability of the model; the backbone network adopts the Focus structure and CSPDarkNet53 to extract multi-level semantic features; the neck network combines the improved fast spatial pyramid pooling structure (Spatial Pyramid Pooling–Fast, SPPF) and the path aggregation network (Path Aggregation Network, PANet) [31] to achieve the full fusion of multi-scale features; the detection head is responsible for outputting the target category and position, and further improves the detection accuracy through the improved loss function and non-maximum suppression strategy. The overall structure of YOLOv5 is shown in Figure 1.

### 2.3. Group-Enhanced Fusion Attention Mechanism

The common channel attention mechanism mainly focuses on the interaction between channels by assigning different weights to each channel to emphasize the channels that contribute most to the task and suppress irrelevant or redundant channels, thereby improving the model’s performance. However, this method often neglects the spatial details within a single channel. When processing the entire feature map, especially when a certain channel is assigned a relatively low overall weight, the spatial information is significantly compressed, resulting in insufficient allocation of importance to different positions in the feature map. To solve this problem, subsequent research introduced the spatial attention mechanism, which weights specific spatial positions in the feature map to highlight the regions that contribute most to the task and suppress irrelevant or redundant regions, thereby improving the model’s performance. However, these methods increase the number of model parameters and computational burden while also causing the attention module to be inefficient and failing to achieve the expected results.

**Figure 2:**
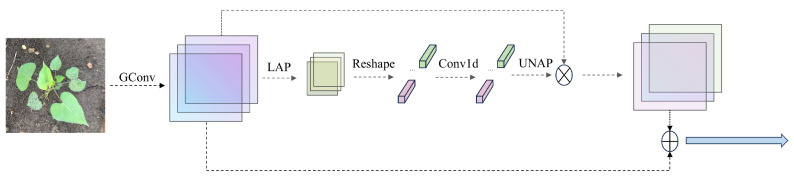
Group enhanced fusion attention mechanism.

To effectively address the current problem of attention mechanisms ignoring spatial features and having high computational costs, this paper proposes a lightweight attention model called Group Enhanced Fusion Attention (GEFA). The core design of GEFA is to achieve more efficient feature extraction through the introduction of group convolution and to fuse additional feature information, thereby achieving lightweight fusion of channel and spatial attention. In GEFA, group convolution uniformly partitions the feature channels into G groups along the channel dimension, where each group is independently convolved. The number of groups is selected to balance computational efficiency and detection accuracy, and all groups maintain equal channel size to ensure stable feature learning. The structure of GEFA is shown in Figure 2, where group convolution allows the network to independently learn features within each feature group, enhancing the model’s ability to recognize the diversity and independence of features. Additionally, inspired by the local SE attention mechanism, this paper further adds local attention processing after group convolution. Specifically, the LAP operation divides the feature map into multiple local regions and performs local average pooling on each region. Then, one-dimensional convolution is used to learn the channel correlation weights between regions, and the weights are restored to the original size through de-pooling to obtain the spatial attention weights for each local region.

(1) $$k=\Phi \left(C\right)={\left|\frac{\log_{2}\left(C\right)}{\gamma }+\frac{b}{\gamma }\right|}_{\mathrm{odd}}$$

The kernel size k corresponds to the number of channels C, indicating that only the local cross-channel interaction information for each channel and its k nearest channels is analyzed. The specific value of k is determined according to the formula. In the formula, C represents the number of channels, k is the value representing the size of the convolution kernel, and γ and b are hyperparameters that need to be set, with a default value of 2. It is required that k be an odd number; if not, it should be incremented by 1. Finally, to capture more comprehensive information, the feature concatenation strategy is adopted, ensuring that the attention module can focus on richer and more comprehensive information. Through such processing, the network not only enhances the feature connections between channels but also finely captures and expresses the local spatial feature information, effectively improving the overall detection performance of the model.

### 2.4. Group-Enhanced Fusion Attention Yolo

**Figure 3:**
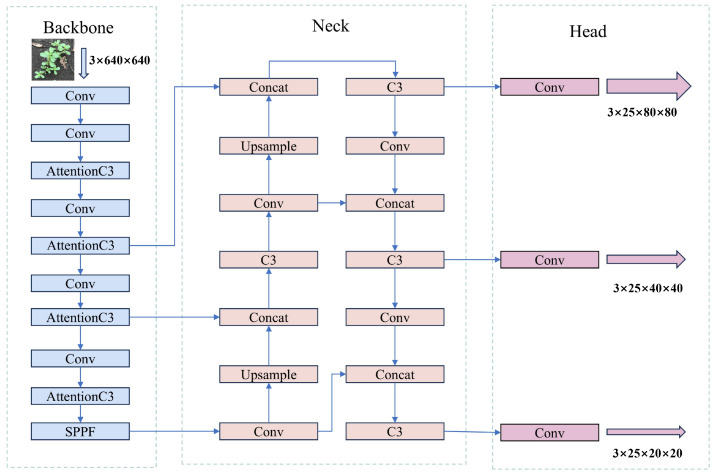
GEFAY network structure.

The YOLOv5 network structure contains multiple C3 structures for image feature extraction. Additionally, the C3 module has multiple functions and uses 1 × 1 convolution and batch normalization layers to effectively reduce the number of parameters and computational complexity of the network. This architecture helps to enhance the depth and width of the network without significantly increasing the computational burden. In the C3 module, the input is divided into two parts, one part is directly passed to the output, while the other part is processed through a series of convolutional layers. This partial skip connection design helps feature fusion while maintaining the depth and complexity of the network, thereby improving information flow. The design of the C3 module enables it to be easily extended to different network architectures and tasks, making YOLOv5 more flexible and effective in handling various object detection tasks. However, although the C3 module can already extract multi-level features, it still has shortcomings in distinguishing the importance of key features. To address this, the GEFA module is introduced on the features output from the C3 module, by effectively weighting the output features, thereby further highlighting important features and improving overall detection performance. Based on this improvement and in combination with YOLOv5s, the Group Enhanced Fusion Attention YOLO (GEFAY) is proposed, and its network structure is shown in Figure 3.

**Figure 4:**
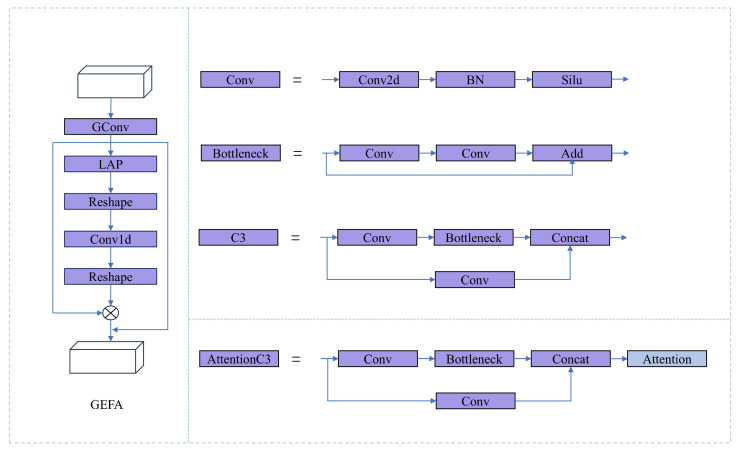
Basic module structure of Group-Enhanced Fusion Attention Yolo.

In this network structure, the input image is first subjected to data augmentation processing, and then the processed image is input into the main network. The main network adopts a strategy combining convolutional layers and the AttentionC3 module to extract image features. The extracted features are transmitted to the PANet of the neck structure at three different scales to achieve multi-level feature fusion; finally, the head performs shape adjustment on the fused features through convolution operations, generating detection outputs at three scales, as shown in Figure 4. The AttentionC3 module mainly consists of two parts: the first part integrates 2D convolutional layers and bottleneck structures and forms a C3 module through residual connections; the second part introduces an attention mechanism and can be flexibly replaced with other attention schemes according to different scenarios.At the same time, the Conv module is composed of 2D convolutional layers, batch normalization layers, and SiLU activation functions, jointly forming the complete feature extraction and processing process.

## 3. Experiments

### 3.1. Experimental Setup and Evaluation Metrics

In this study, three main datasets were used for model training and validation: CottonWeedDet12 [32], which was specifically designed for the detection of cotton weeds in agricultural scenarios, and the generalization test datasets Pascal VOC 2012 (VOC) [33] and Common Objects in Context 2017 (COCO) [34].

**Figure 5:**
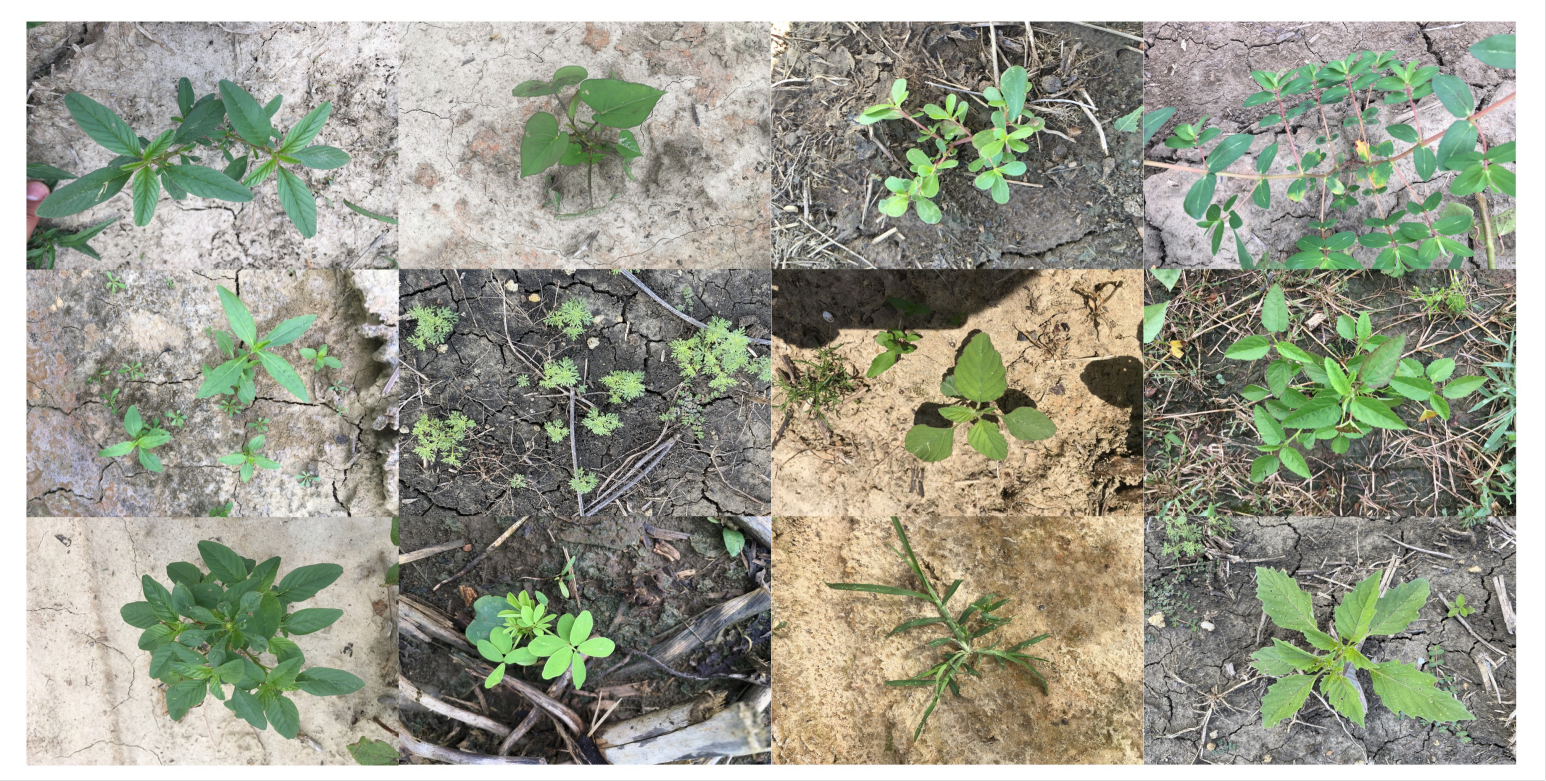
Sample images of the CottonWeedDet12 dataset.

CottonWeedDet12 is a dedicated image dataset constructed for the task of detecting weeds in cotton fields in agricultural scenarios. It contains 5648 high-resolution images collected in real field environments, covering 12 types of typical weeds such as purslane, cowpea grass, licorice plant, ragweed, and morning glory. To ensure the balanced distribution of data during the training, validation, and testing phases, it was divided into training set, validation set, and test set in a ratio of 7:2:1. Examples of weeds in some of the datasets are shown in Figure 5. This dataset ensures the accuracy of spatial positioning information through a standardized annotation process. Its multi-category coverage, diverse lighting conditions, and plant growth stage characteristics provide a reliable basis for evaluating model accuracy and generalization ability.

**Figure 6:**
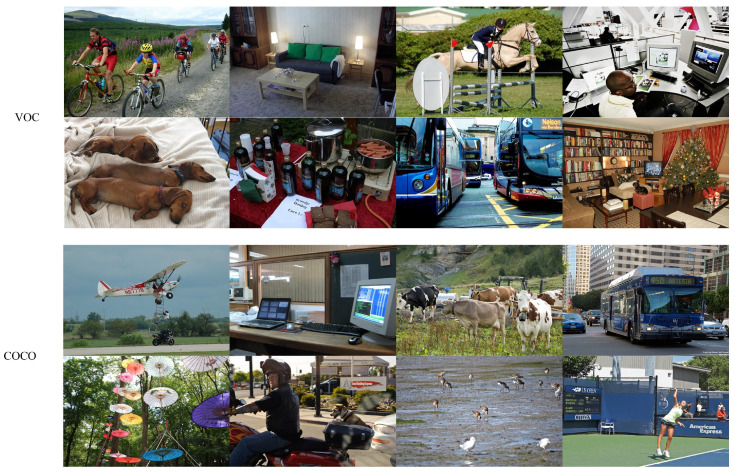
Sample images of the VOC and COCO datasets.

The Pascal VOC 2012 dataset includes 20 categories, while COCO 2017 contains 80 categories, with example images shown in Figure 6. In the VOC 2012 dataset, the training set consists of 13,700 photos, and the test set includes 3425 images. For the COCO 2017 dataset, the training set contains 118,287 images, and the test set has 5000 images. During the training process, the image size was set to 640 × 640 pixels. The training lasted for 300 cycles, with a batch size of 40 for each iteration, a learning rate of 0.01, and a momentum of 0.937. The early stopping mechanism was adopted (with a waiting time of 100), and the default optimizer used was SGD. The experimental setup used in this study is outlined in Table 1.

The performance evaluation of the object detection network is primarily based on the mean Average Precision (mAP) during the training phase and its performance on the validation set. To accurately measure detection effectiveness, precision, recall, and mAP are used as key performance evaluation metrics. Additionally, the formulas for calculating precision and recall are described as follows:

(2) $$\mathrm{Recall}=\frac{TP}{TP+FN}$$

(3) $$\mathrm{Precision}=\frac{TP}{TP+FP}$$

True Positives (TP): The number of positive samples correctly classified as positive by the classifier. True Negatives (TN): The number of negative samples correctly identified as negative by the classifier. False Positives (FP): The number of negative samples incorrectly classified as positive by the classifier. False Negatives (FN): The number of positive samples incorrectly classified as negative by the classifier. Average Precision (AP) is the area under the Precision-Recall (P-R) curve. When evaluating network performance, the higher the AP, the better the performance. Calculating the AP for each category and then taking the average yields the Mean Average Precision (mAP), which is a comprehensive measure of the object detection network. mAP0.5 indicates that the AP for each category is calculated at an Intersection over Union (IoU) threshold of 0.5. By varying IoU thresholds from 0.5 to 0.95 in increments of 0.05, the metric mAP0.5:0.95 is obtained.

In addition to detection accuracy, computational efficiency plays a crucial role in object detection models, especially for real-time applications. Computational performance is evaluated through FLOPs, GPU inference latency (ms), FPS, and parameter count. Latency (ms) represents the average time required for a single forward inference on the GPU, reflecting the responsiveness of the model, while FPS denotes the number of images processed per second and directly indicates real-time capability. Lower latency corresponds to faster single-frame processing, and higher FPS implies better real-time performance. The frames per second (FPS) in the data report sometimes does not include preprocessing and post-processing operations such as Non-Maximum Suppression (NMS).

### 3.2. Comparative Experiments

To verify the effectiveness of the proposed Group Enhancement Fusion Attention Mechanism (GEFA) in the weed detection task, experiments were conducted based on the CottonWeedDet12 dataset of cotton fields, and a comparative analysis was carried out with various common attention mechanisms.

To ensure the consistency of the experiments, the GEFA and the attention mechanisms such as SE, CBAM, ECA, and CA [35] were integrated into the same network framework. Different attention structures were compared by replacing the AttentionC3 module in the GEFAY network. The selected attention mechanisms are all representative: SE represents the typical channel attention mechanism, ECA represents the lightweight channel attention mechanism, CA embodies the coordinate attention idea with position information perception ability, and CBAM is the classic representative of channel and spatial joint attention; additionally, MLCA represents the multi-scale/composite attention structure, and GEFA embodies the design concept of enhanced fusion attention. To ensure fairness, a dimension compression strategy was adopted for some attention structures with large parameter quantities. The denominator represents the dimension reduction factor, and the numbers in parentheses represent the convolution kernel size in the convolution, as shown in Table 2. It is worth noting that even without dimension reduction, GEFA can maintain a high detection performance while only causing a very small increase in model complexity, demonstrating a good balance between efficiency and effectiveness.

Table 3 presents the experimental results of different attention mechanisms on this dataset, including mAP@0.5, model parameters, GFLOPS, and GPU inference speed. By comparing, it can be observed that the GEFA attention mechanism performs exceptionally well across multiple metrics, and it can effectively enhance the target detection performance while maintaining the model’s lightweight nature. In terms of the mAP@0.5 metric of the model, the network with the GEFA attention mechanism performs the best, reaching 95.0%, while networks with other attention mechanisms perform similarly but slightly less well. For example, the mAP@0.5 of the networks with SE/4 and CBAM/16 is both 94.0%, while ECA’s mAP@0.5 is 94.6%, and CA/16 is 94.3%. Under the stricter evaluation criterion of mAP@0.5:0.95, GEFAY also leads the way, reaching 87.0%, surpassing other methods, with the closest being CA, whose mAP@0.5:0.95 is 85.7%. Thus, GEFA can achieve significant performance improvements across multiple mAP standards.

**Figure 7:**
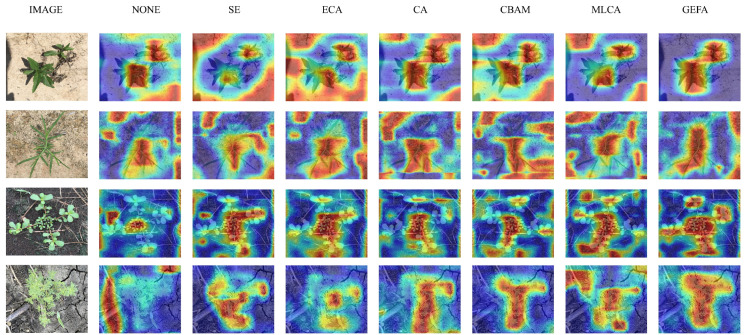
Comparison of heat maps under different attention mechanisms by GEFA.

To further verify the effectiveness of the GEFA attention mechanism, this study employed the Grad-CAM++ [36] method togenerate heat maps that included different attention mechanisms (such as SE, ECA, CA, CBAM, MLCA, and GEFA), and compared their performance in the weed detection task. The heat maps are shown in Figure 7, which visually illustrate the activation distribution of each network in the input image for the target area. From the figure, it can be seen that the GEFAY network shows stronger focus on the concentrated area in the heat map. This indicates that GEFAY can more accurately focus on the details of the weed target and capture key edge information, solving problems of complex types and variable shapes.

**Figure 8:**
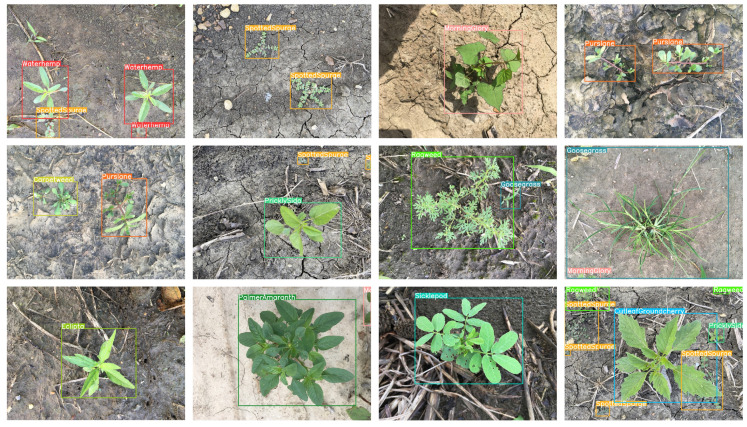
The visual detection results of GEFAY in cotton weed detection.

Based on the above experimental results, it can be concluded that the GEFA attention mechanism performs exceptionally well in the cotton weed detection task, helping GEFAY achieve the highest accuracy while maintaining the network’s computational efficiency and inference speed, thereby effectively enhancing the model’s adaptability to complex scenarios. Figure 8 shows the visual detection results of GEFAY in cotton weed detection.

The enhanced network of GEFA can accurately distinguish between cotton and weeds, and precisely locate the positions of the weeds, demonstrating the advantage of GEFA in capturing details. Based on the above experimental results, it can be concluded that the GEFA attention mechanism performs exceptionally well in the cotton weed detection task, helping GEFAY achieve the highest accuracy while maintaining the network’s computational efficiency and inference speed, effectively enhancing the model’s adaptability to complex scenarios.

### 3.3. Verification of Cross-Scene Generalization Capability

To further verify the effectiveness of GEFA and GEFAY, experiments were conducted on the VOC and COCO datasets. This is because these two datasets are benchmark datasets in the field of computer vision, possessing wide representativeness and challenges. Additionally, conducting experiments on the VOC and COCO datasets helps ensure the model’s cross-scenario generalization ability. The images in these datasets come from various different environments and backgrounds, which can simulate complex detection scenarios in the real world, thereby helping to evaluate the stability and robustness of the GEFA mechanism under different conditions. Through training and testing on these diverse datasets, it can provide more solid support for subsequent applications in various weed detection tasks.

**Figure 9:**
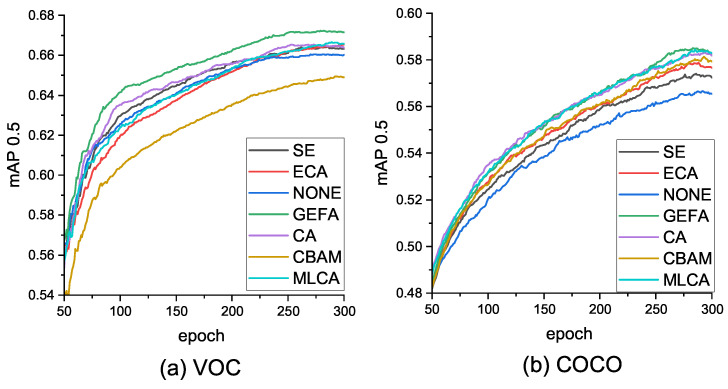
Comparison of GEFA with other attention mechanisms in the mAP@0.5 metric.

Figure 9 displays the comparison of mAP@0.5 for various attention mechanisms on the VOC and COCO datasets. “NONE” indicates the GEFAY model without any additional attention mechanisms, while others represent the addition of that attention method to the GEFAY network. To enhance the persuasiveness of the comparative experiments, we adjusted the channels of the attention mechanisms to maintain a roughly consistent scale. Observing Figure 9, it is evident that our proposed GEFA attention mechanism is more effective.

The model parameters, computational cost, and network speed on the VOC and COCO datasets are shown in Table 4 and Table 5 respectively. The proposed GEFA method only slightly increases the network size but significantly improves the mAP value of the network, thereby achieving lightweight and effective enhancement of network performance. Specifically, on the VOC dataset (Table 4), the mAP@0.5 of GEFA reaches 67.1%, the mAP@0.5:0.95 reaches 46.5 which are respectively 0.6 and 2.6 percentage points higher than the baseline model, and GFLOPS slightly increases to 17.6, while the GPU inference speed remains at 9.6 ms. Compared with methods such as SE/4 and CBAM/16, it still has a better balance. On the COCO dataset (Table 5), the mAP@0.5 of GEFA is 58.5%, the mAP@0.5:0.95 is 38.2%, also surpassing other attention mechanisms, and the inference speed still remains at around 9.6 ms, indicating that with a slight increase in network complexity, significant performance improvement can be achieved. Compared with other attention methods, the model with GEFA added performs better with similar complexity.

**Figure 10:**
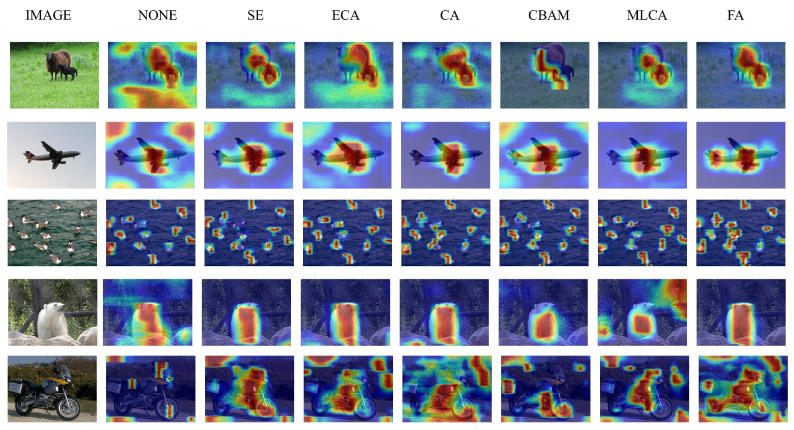
Comparison of heatmap experimental results for different attention mechanisms.

Figure 10 presents the visualized results of Grad-CAM++ heatmaps generated by the GEFAY network integrating different attention mechanisms on the VOC and COCO datasets. These results clearly illustrate the differences in the ability of each attention method to focus on the target area.

**Figure 11:**
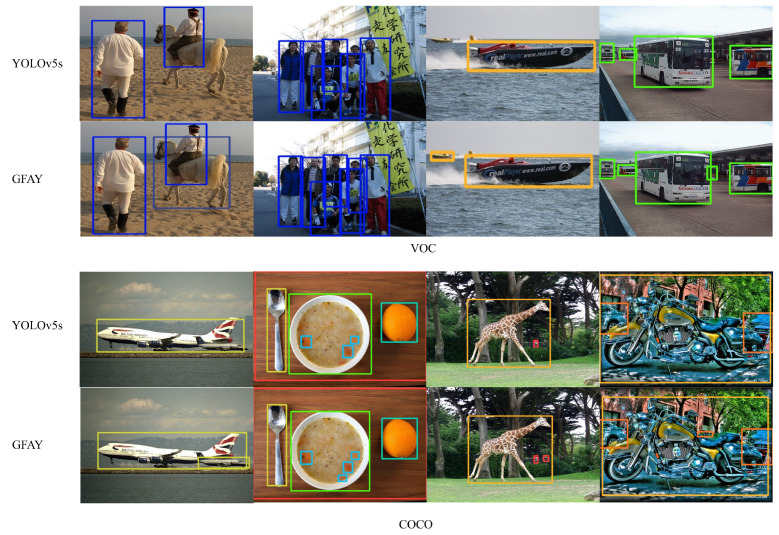
Comparison of detection results between YOLOv5s and GEFAY.

To further examine the effectiveness of our proposed attention mechanism, we tested the performance of our model on the VOC and COCO datasets, as shown in Figure 11, where it displayed better results in complex environments involving small targets and occlusions.

### 3.4. Performance Evaluation of Object Detection Algorithms

To further evaluate the robustness and generalization capability of the proposed GEFAY model beyond the weed detection task, additional experiments were conducted on the SSDD [37] and NEU-DET [38] datasets. These datasets were employed to examine whether the lightweight attention refinement designed for weed recognition can consistently enhance feature representation under different object scales and background complexities. In these experiments, GEFAY was compared with several representative object detection methods, including Faster R-CNN [39], YOLOv5s, YOLOv7-tiny [40], and YOLOX [41], all implemented using the official open-source frameworks provided by the original authors. The quantitative results are summarized in Table 6 and Table 7.

**Figure 12:**
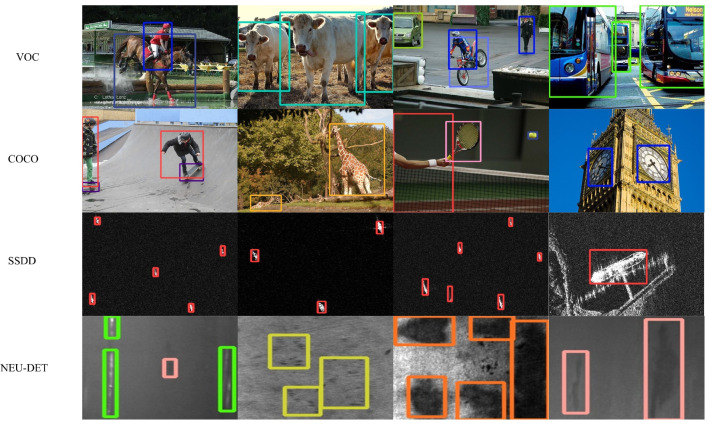
Some detection results of GEFAY on various datasets.

The qualitative results of the GEFAY algorithm proposed in this paper on four datasets are shown in Figure 12. From the figure, it can be seen that the GEFAY model is more effective at bounding various corresponding targets, and it still performs well even in complex environments.As shown in the results, although GEFAY is optimized primarily for weed detection under real agricultural conditions, it maintains competitive detection accuracy on industrial datasets while preserving a lightweight model structure. This demonstrates that the proposed attention refinement does not overfit to a specific weed dataset but instead improves feature extraction in a task-driven and deployment-oriented manner. Such robustness is essential for practical agricultural applications, where background variability and target appearance can differ significantly across real-world scenarios.

## 4. Design and Implementation of Weed Detection System

### 4.1. Research Motivation

The traditional methods for identifying weeds rely on manual inspection and mechanical weeding. This process is not only time-consuming and labor-intensive, but also easily affected by weather and environmental factors, resulting in low efficiency and limited detection accuracy. Although there are many types of existing weed detection technologies, most of them fail to balance automation, real-time performance, and detection accuracy, and thus have limitations. The traditional detection process usually requires users to manually complete scattered steps such as image acquisition, model operation, and result viewing, lacking an integrated operation interface. To solve this problem, it is urgent to develop an automated weed detection system that integrates multiple models [42]. This device system will integrate image acquisition, weed detection, result display, etc., providing a unified and intuitive user interface, thereby avoiding frequent window switching or cumbersome manual operations, and significantly improving work efficiency. Moreover, by burning the YOLO model onto the development board [43], the usability of the model on the embedded platform has been achieved. The system equipment automatically analyzes farmland images through intelligent algorithms, accurately identifies the types and distribution of weeds, provides data support for precise weeding decisions, and simultaneously enhances the automation and intelligence levels of agricultural management. The development of this system will greatly enhance the efficiency and accuracy of weed detection, thereby promoting the sustainable development of agricultural production.

### 4.2. Design of Weed Detection System

The research objective of this system is to develop a simple and real-time weed detection system suitable for precision agriculture scenarios. The system should meet the following requirements: (1) support flexible configuration of different detection models and parameters to adapt to different farmland environments and detection needs; (2) support two application modes: batch image detection and real-time video detection; (3) possess good human-computer interaction capabilities and result visualization capabilities.

This system mainly includes three core modules: parameter configuration module, data selection module, and result display module.

1. **Parameter Configuration Module**: After the model selection is completed, the system will guide the user into the parameter configuration stage. The core of this stage lies in adjusting key hyperparameters to adapt to different detection difficulties, target densities, and equipment performance constraints.
2. **Data Selection Module**: Given that weed detection tasks may present multiple input forms in practice, the system has designed multiple data input methods for the detection phase to meet the different needs and operating habits of users.
3. **Result Display Module**: After the weed detection process is completed, the system will visualize the results obtained from the analysis, so that users can intuitively observe the distribution of weeds in the screen.

**Figure 13:**
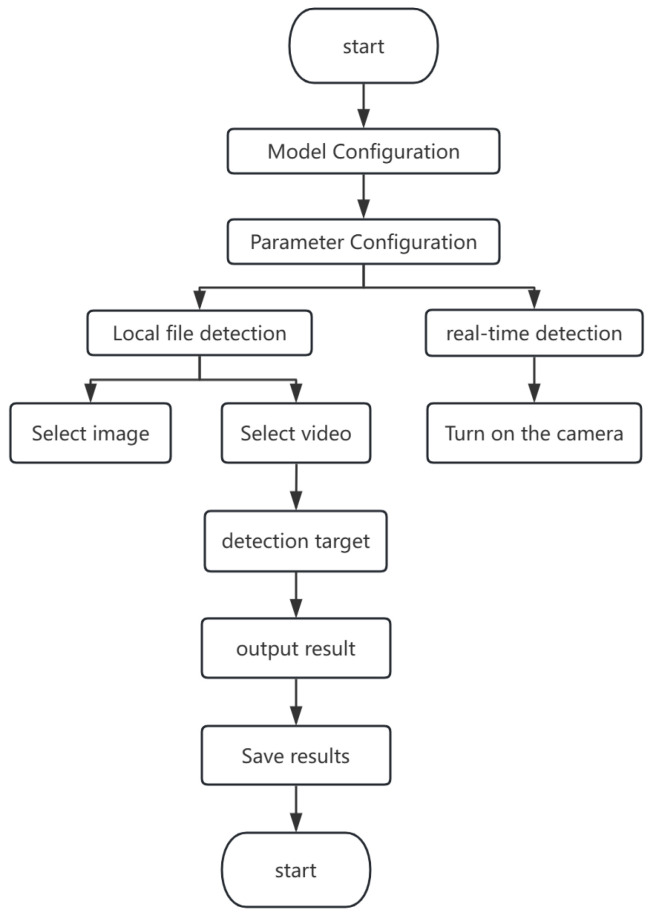
Design process of detection system.

The overall process of the weed detection system is shown in Figure 13. The system first allows users to select different models and parameter configurations to meet different detection requirements. Users can upload local files for batch detection or choose the real-time monitoring mode to identify weeds in the video stream. Through precise algorithms, the system can automatically identify weeds in two modes and output detection results.

**Figure 14:**
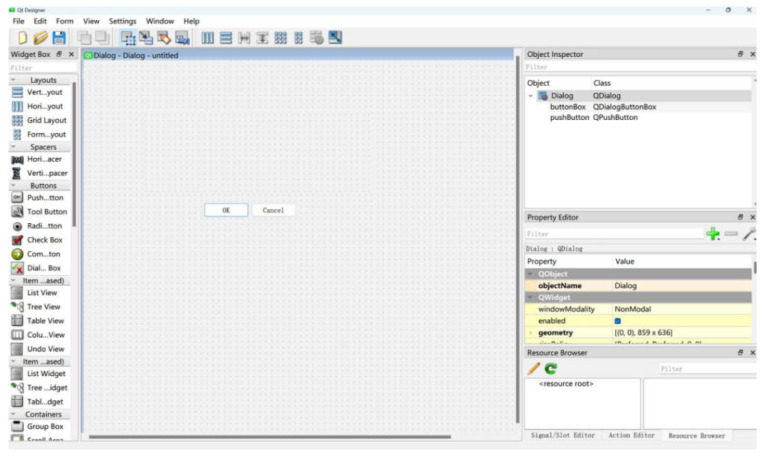
Qt Designer design interface.

To ensure consistency in development and deployment between modules, this weed detection system uses Python as the core development language. The main reason is the richness of its deep learning and computer vision ecosystem, as well as its high compatibility with mainstream machine learning frameworks. The system adopts a modular design and reserves standardized interfaces for subsequent algorithm optimization and functional expansion. The development environment uses PyQt5 as the graphical user interface framework to achieve good support for multiple platforms and different configuration environments. In PyQt5, QtDesigner is a graphical interface design tool designed to simplify the interface development process. Figure 14 shows the Qt Designer design interface.This system was implemented and tested under the following hardware and software environments, as shown in Table 8.

### 4.3. Implementation of Weed Detection System

#### 4.3.1. Parameter Configuration Module

**Figure 15:**
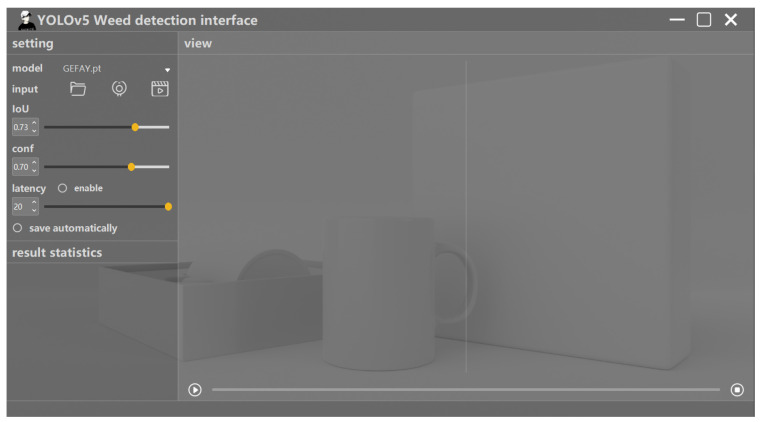
The functional demonstration of the parameter configuration module.

In this stage, the system enables users to flexibly adjust key parameters according to specific detection scenarios, including IoU threshold, confidence threshold, and system response delay. Users can also enable the auto-save function to ensure that the test results are stored in a timely manner, facilitating subsequent data analysis and processing. Through this parametric design, the system can achieve adaptive adjustment in different application environments and optimize the overall detection performance. As shown in Figure 15, the specific contents of the adjustable parameters of the system are presented.

#### 4.3.2. Data Selection Module

**Figure 16:**
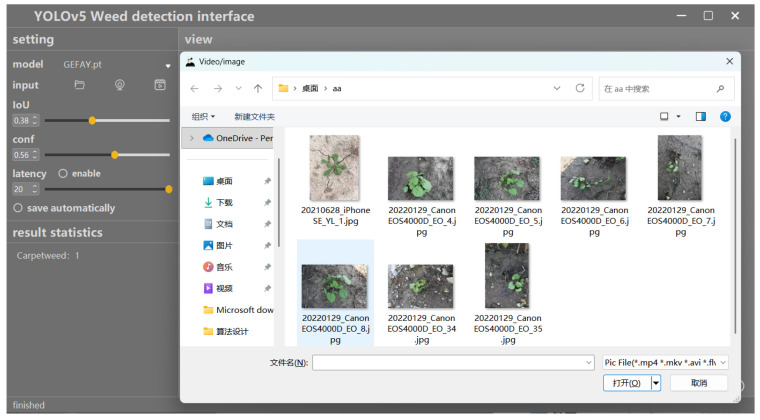
Data sources selection module.

This module provides two detection methods for users to choose from: one is an offline detection mode based on file upload, which supports batch processing of image and video files; the second is the real-time detection mode, which directly calls the camera to obtain the video stream and achieve real-time weed recognition. Users can choose the most suitable method based on their actual needs and operating environment to ensure that the detection process is both efficient and accurate. Figure 16 shows the process of selecting input files in the system data selection module.

#### 4.3.3. Result Display

**Figure 17:**
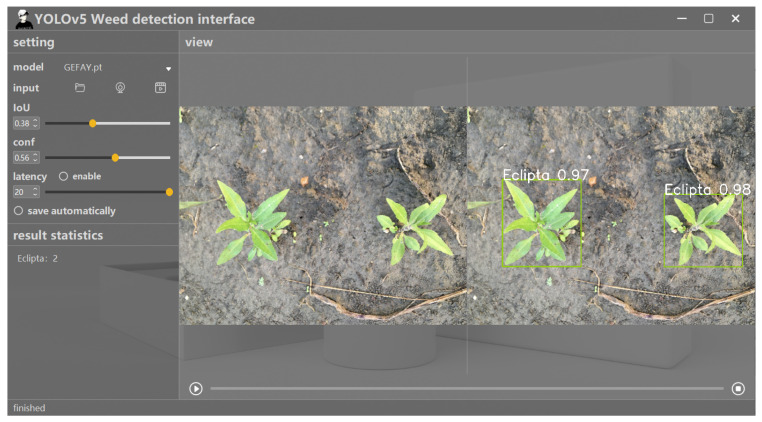
The functional demonstration of the result display module.

After the detection is completed, the system will visually present the target detection results. Each detection object is marked with a border and accompanied by a category and confidence score for users to quickly evaluate detection accuracy. This module adopts an intuitive graphical interface design, supporting users in zooming in and performing other operations on the detection results, further improving the readability and practicality of the data. The detection results of the system are shown in Figure 17.

### 4.4. Deployment of Weed Detection System Equipment

#### 4.4.1. Introduction to Edge Devices

**Figure 18:**
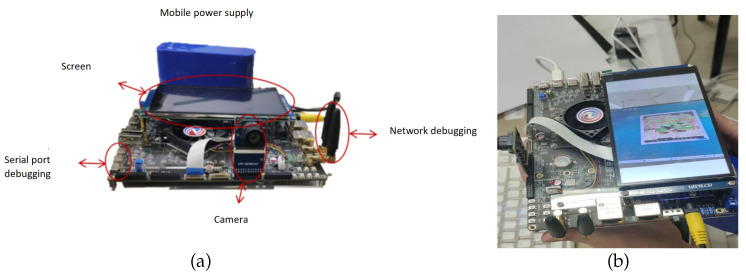
(**a**) equipment deployment; (**b**) another equipment deployment.

This research is oriented toward the development needs of agricultural intelligence. Its core objective is to design a weed recognition model that combines efficient computing performance, precise recognition ability, and lightweight architecture, laying a solid foundation for the subsequent deployment of embedded platforms. The trained GEFAY weight file is converted into a format to generate the RKNN format suitable for embedded hardware operation, and finally, the deployment and verification on the RK3588 development board are completed. RK3588 selected for the deployment of the development board, after careful screening in the hardware configuration, fully meet the demand of the real time and stability of weed identification scenarios.This development board is equipped with a 5.5-inch MIPI interface 1080p high-definition display screen, which can clearly present the image data and recognition results during the weed detection process, facilitating the intuitive observation and interaction of operators. It also integrates the MCIMX415 professional camera (Zengding Atomic, Dongguan, China), which has high-resolution imaging capabilities and provides high-quality input image data for the model. More importantly, the RK3588 development board features outstanding image processing hardware performance, supporting 8K video decoding at 60 frames per second. This core characteristic enables the development board to achieve smooth real-time computing and analysis when processing high-speed collected field image data, effectively avoiding recognition lag caused by data processing delays. This deployment fully meets the need for real-time weed identification in agricultural field operations. It not only verifies the practicality of the model on embedded platforms but also demonstrates its scalability in practical applications. The deployed device and its detection results are shown in Figure 18 and Table 9.

#### 4.4.2. The Identification Process of Equipment Modules

As shown in Figure 18 andFigure 19, the system enables users to upload local images through the graphical interface for weed identification. After the input image undergoes preprocessing operations such as size normalization and format conversion, it is sent to the GEFAY model for inference calculation. The model outputs the category information of the weed targets and presents the detection results in the form of bounding boxes and confidence levels. The identification results can be displayed on the computer interface, facilitating users to visually view and analyze them.

Furthermore, by calling the camera to obtain consecutive image frames in embedded devices, continuous recognition of weeds in the field and continuous update of results can be achieved, which further enhances the applicability and practical value of the system in actual agricultural operation scenarios. This overall process realizes an end-to-end closed loop from image acquisition and model inference to result visualization, laying a solid foundation for subsequent embedded deployment and on-site application.

Table 10 compares the FPS of the proposed system under online PC-based inference and embedded edge deployment, reflecting both inference efficiency and end-to-end real-time performance.

**Table 9:** Embedded platform specifications.

Platform	Specification
Embedded Board	Rockchip RK3588 Development Board
CPU	8-core heterogeneous architecture: 4 × Cortex-A76 + 4 × Cortex-A55, up to 2.4 GHz
NPU	Built-in Neural Processing Unit (NPU), peak performance up to 6 TOPS
Camera Module	MCIMX415 high-resolution industrial camera
Display	5.5-inch MIPI display, 1080p resolution

**Table 10:** FPS comparison between online system and edge device deployment.

Deployment Scenario	Platform	Input Resolution	FPS
Online system	PC (RTX 3090)	640 × 640	310.9
Edge deployment	RK3588	640 × 640	29.19

**Figure 19:**
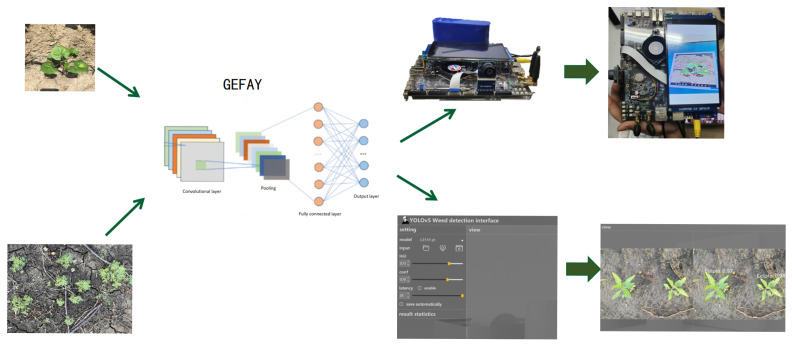
Equipment deployment.

### 4.5. Chapter Summary

This chapter presents a weed detection system based on the YOLO model and PyQt5, which supports the GEFAY model and can adaptively adjust the IoU and confidence threshold. It is also compatible with local file and camera detection, and provides visual output of box selection annotations and confidence scores. The system adopts a modular design, which not only enhances the accuracy and real-time performance of agricultural scene detection, but also reserves standardized interfaces to support algorithm optimization and functional expansion, providing an operational technical platform for intelligent management and precise weeding decisions.

## 5. Conclusions

In response to the challenges in cotton field weed management under the context of precision agriculture, such as the presence of multiple weed species, complex background, and the need for model deployment on embedded terminals, this paper proposes a lightweight attention mechanism named “Grouped Enhanced Fusion Attention” (GEFA). This mechanism effectively integrates channel and spatial cues by combining group convolution with one-dimensional convolution, addressing the problem that channel attention mechanisms often ignore spatial information, while spatial attention mechanisms have relatively high computational costs. Based on GEFA, this paper embeds GEFA into the YOLOv5 framework to construct the GEFAY object detection network. Experiments on the CottonWeedDet12 dataset show that GEFA/GEFAY can improve weed detection accuracy while only causing a moderate increase in parameters and computational costs, thus achieving a good balance between accuracy and efficiency in field applications. Further evaluations on VOC and COCO further confirm that the proposed attention refinement method can provide consistent improvements in various scenarios, thereby providing robust support for real agricultural environments. In addition to algorithm verification, an end-to-end weed detection system with visualization and interaction capabilities, which supports image inference and detection based on real-time cameras, has been implemented. Deployment on the RK3588 edge platform has confirmed the practicality of the proposed method under resource-constrained conditions. We plan to further explore the integration of GEFA with image classification and semantic segmentation networks in the future and deploy it on a wider range of embedded devices, as well as apply it to various computer vision tasks.

## Figures and Tables

**Table 1:** Experimental platform specifications.

Platform	Specification
CPU	Intel(R) Xeon(R) Gold 6330 CPU
GPU	NVIDIA GeForce RTX 3090
Operating System	Ubuntu 18.04.5 LTS
Framework	PyTorch 1.13

**Table 2:** Different settings of dimensionality reduction factors for attention mechanisms and corresponding model parameters.

Attention	GFLOPS	Parameters (m)
NONE	16.1	7.07
SE/4	17.4	7.38
ECA	17.4	7.37
CA/16	17.4	7.39
CBAM/16	17.4	7.37
MLCA (5 × 5)	17.5	7.35
MLCA (9 × 9)	17.8	7.42
GEFA (5 × 5)	17.6	7.39
GEFA (9 × 9)	17.9	7.43

**Table 3:** The experimental results on the CottonWeedDet12 dataset.

Attention Mechanism	mAP@0.5	mAP@0.5:0.95	Parameters (M)	GFLOPs	GPU Speed (ms)	FPS
None	94.0%	84.6%	7.04	15.9	6.5	353.8
SE/4	94.0%	84.0%	7.35	17.2	9.7	337.6
ECA	94.6%	85.3%	7.36	17.3	9.1	282.4
CA/16	94.3%	85.7%	7.36	17.3	11.1	299.1
CBAM/16	94.2%	84.4%	7.35	17.3	10.8	325.7
MLCA (5×5)	94.8%	85.3%	7.34	17.3	10.6	177.7
GEFA (5×5)	95.0%	87.0%	7.36	17.5	9.6	310.9

**Table 4:** Comparison of experimental results of different attention mechanisms on the VOC dataset.

Attention Mechanism	mAP@0.5	mAP@0.5:0.95	Parameters (M)	GFLOPS	GPU Speed (ms)
NONE	66.5%	43.9%	7.07	16.1	6.5
SE/4	66.3%	44.6%	7.38	17.4	9.7
ECA	66.5%	44.7%	7.37	17.4	9.1
CA/16	66.5%	44.9%	7.39	17.4	11.1
CBAM/16	64.9%	43.0%	7.37	17.4	10.8
MLCA (5 × 5)	66.6%	44.8%	7.35	17.5	10.6
GEFA (5 × 5)	67.1%	46.5%	7.39	17.6	9.6

**Table 5:** Comparison of experimental results of different attention mechanisms on the COCO dataset.

Attention Mechanism	mAP@0.5	mAP@0.5:0.95	Parameters (M)	GFLOPS	GPU Speed (ms)
NONE	56.7%	36.5%	7.23	16.6	6.6
SE/4	57.3%	37.3%	7.54	17.9	9.9
ECA	57.7%	37.5%	7.53	17.9	9.3
CA/16	58.2%	38.1%	7.55	17.9	11.4
CBAM/16	57.7%	37.6%	7.54	17.9	10.7
MLCA (5 × 5)	58.3%	38.0%	7.52	18.0	10.4
GEFA (5 × 5)	58.5%	38.2%	7.55	18.1	9.3

**Table 6:** Comparison of the GEFAY algorithm with other algorithms on the SSDD dataset.

Model	mAP@0.5	mAP@0.5:0.95	Parameters (M)	GFLOPS	GPU Speed (ms)
Faster RCNN	87.70%	-	78.1	41.1	20.4
YOLOX-s	90.28%	60.21%	8.94	26.8	15.5
YOLOv5-s	98.33%	69.32%	6.58	15.8	6.2
YOLOv7-tiny	97.52%	66.91%	5.73	13.0	3.6
GEFAY (Ours)	98.43%	71.24%	7.33	17.3	9.4

**Table 7:** Comparison of the GEFAY algorithm with other algorithms on the NEU-DET dataset.

Model	mAP@0.5	mAP@0.5:0.95	Parameters (M)	GFLOPS	GPU Speed (ms)
Faster RCNN	67.80%	-	41.2	10.5	12.3
YOLOX-s	67.63%	33.78%	8.94	3.3	4.6
YOLOv5-s	75.03%	37.72%	6.70	15.8	5.2
YOLOv7-tiny	73.12%	35.81%	5.74	13.1	3.9
GEFAY (Ours)	75.82%	42.01%	7.34	17.5	9.3

**Table 8:** Local experimental environment configuration.

Platform	Specification
CPU	Intel(R) Core(TM) Ultra 5 225H (1.70 GHz)
GPU	Intel(R) Arc(TM) 130T GPU(16GB)
Operating System	Microsoft Windows 11
GUI Framework	PyQt5 5.15.14

## Data Availability

All of the data come from open-source datasets and are publicly accessible. Also, the datasets are available from the First and Corresponding authors on request.

## Abbreviations

The following abbreviations are used in this manuscript:

GEFAY	Group-Enhanced Fusion Attention YOLO
GEFA	Group-Enhanced Fusion Attention
